# Observational Skill-based Clinical Assessment tool for Resuscitation (OSCAR): Development and validation^☆^

**DOI:** 10.1016/j.resuscitation.2011.03.009

**Published:** 2011-07

**Authors:** S. Walker, S. Brett, A. McKay, S. Lambden, C. Vincent, N. Sevdalis

**Affiliations:** a Department of Surgery & Cancer, Imperial College London, St Mary's Hospital Campus, 10th Floor QEQM Building, St Mary's Hospital, Praed Street, London W2 1NY, UK; b Centre for Perioperative Medicine and Critical Care Research, Department of Anaesthesia and Intensive Care, Hammersmith Hospital, Imperial College Healthcare NHS Trust, Du Cane Road, London W12 0HS, UK; c Department of Resuscitation and Outreach, St Mary's Hospital, Imperial College Healthcare NHS Trust, Praed Street, London W2 1NY, UK; d Department of Anaesthetics, University College London Hospitals NHS Trust, 235 Euston Road, London, NW1 2BU, UK; e Clinical Safety Research Unit, Department of Surgery & Cancer, Imperial College London, 10th Floor QEQM Building, St Mary's Hospital, Praed Street, London W2 1NY, UK

**Keywords:** Non-technical skills, Teamworking skills, Adverse events, Cardiac arrest teams, Resuscitation training, Assessment tool

## Abstract

**Aim:**

The aim of the study reported here was to address the need to assess and train teamwork and non-technical skills in the context of Resuscitation. Specifically, we sought to develop a tool that is feasible to use and psychometrically sound to assess team behaviours during cardiac arrest resuscitation attempts.

**Methods:**

To ensure validity, reliability, and feasibility, the Observational Skill based Clinical Assessment tool for Resuscitation (OSCAR) was developed in 3 phases. A review of the literature leading to initial tool development was followed by an assessment of face and content validity, and finally a thorough reliability assessment, using Cronbach's α to assess internal consistency and intraclass correlation to assess inter-rater reliability.

**Results:**

OSCAR was developed methodically, and tested for face and content validity. Cronbach's α results ranged from 0.736 to 0.965 demonstrating high internal consistency, and intraclass correlation results ranged from 0.652 to 0.911, all of which are strongly significant and indicate good inter-rater reliability.

**Conclusion:**

On the basis of our results, we conclude that OSCAR is psychometrically robust, scientifically sound, and clinically relevant. We have developed the Observational Skill-based Clinical Assessment tool for Resuscitation (OSCAR) for the assessment of non-technical skills in Resuscitation teams. We propose the use of this tool in simulation and real Cardiac Arrest Resuscitation attempts to assess, guide and train non-technical skills to team members, to improve patient safety and maximise the chances of successful resuscitation.

## 1 Introduction

Effective resuscitation requires a combination of good technical and non-technical skills to ensure safe and efficient task performance. ‘Non-technical skills’ are skills complementary to a clinician's technical ability. They include communication, decision making, leadership, task management and monitoring^1–5^ and are critical to effective teamwork.^6,7^ To date, non-technical skills have been relatively over-looked in healthcare, with an emphasis on training the technical aspects of various tasks. This is, however, beginning to change in light of various reports^8,9^ identifying the incidence of error and adverse events in hospitals, and the fact that there is often a failure in team-working skills and communication as contributing factors. Evidence shows that failure in these skills has an impact on safety of care and overall patient outcomes by influencing teamwork, coordination of care, and the efficiency of care provided.^1^ The current consensus is that approximately 10% of hospital inpatients are likely to suffer an adverse event, of which half are considered preventable.^10^

The specialties of critical care and anaesthesia have followed the trend of emphasising the importance of patient safety and the role of non-technical skills in adverse events in healthcare.^1,6,11^ In 2009, The European Society of Intensive Care Medicine launched “*Patient safety in intensive care medicine: the Declaration of Vienna*” ^12^ with the aim of raising the profile of patient safety and quality of care issues, and supporting research into this area of healthcare. The declaration concludes that “*a significant number of dangerous human errors occur in the ICU. Many of these errors can be attributed to problems of communication between the physicians and nurses. Applying human factor engineering concepts to the study of the weak points of a specific ICU may help to reduce the number of errors”* (p. 1670). In addition, the Helsinki Declaration on Patient Safety in Anaesthesiology ^13^ published in June 2010 also endorses non-technical skills training as a key component of improving patient safety.

Care of a patient in the emergency setting is particularly prone to errors and adverse events. Various studies^14,15^ have noted a higher rate of adverse events during emergency resuscitation (whether medical or trauma care) compared with the general hospital population. This is attributable to many factors, including the increased rate of patient interventions, the time-critical nature of care, the need for rapid decision-making often with limited patient information, and the fact that “teams” are assembled instantly by the emergency call. These *ad hoc* team members may have never worked together before or even met each other. All of these factors support the need to improve an awareness and training of non-technical skills for emergency team members.

To facilitate effective training in non-technical skills, a reliable tool is required, which captures these skills robustly, can be used to identify strengths and weaknesses, and also to facilitate systematic, constructive feedback. To date, whilst various tools have been developed to assess non-technical skills in operating theatre environments,^3,16,17,18^ no tool exists specifically to measure the performance of individual team members within a resuscitation context. This means that whilst the technical skills of resuscitation can be assessed and trained, teamwork and non-technical skills may be neglected. In addition to skills assessment and feedback, a further benefit of such a tool would be in the evaluation of the human factors impact of proposed developments in resuscitation, be they novel procedures or items of equipment.^7^

The aim of the study reported here was to develop and verify the “Observational Skill-based Clinical Assessment tool for Resuscitation” (OSCAR) tool, which measures the non-technical skills of resuscitation team members.

## 2 Methods

**Fig. 1:**
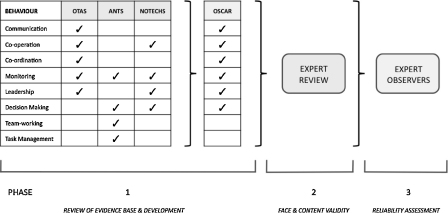
Methodology and phases of development of OSCAR.

To ensure validity, reliability, and feasibility, OSCAR was developed in three phases (Fig. 1).^19^

### 2.1 Phase 1 – review of evidence base, and initial tool development

There are a number of non-technical skills assessment tools published in the context of surgery and anaesthesia, but none are directly applicable to resuscitation. We chose three tools of relevance as a starting point for our study. These were the Observational Teamwork Assessment for Surgery (OTAS),^16^ anaesthetists’ non-technical skills (ANTS),^3^ and the revised NOn-TECHnical skills (NOTECHS) scale for operating theatres.^17^ These tools measure non-technical skills either for individual team-members (ANTS; NOTECHS), or for the entire team (OTAS), and have been shown to capture these skills in real-time observation in clinical environments, and in simulation-based training modules.^3,4,20,21^ Whilst the behaviours measured are given slightly different terms in each of the tools, broadly very similar assessments are made.

Building on this evidence base, OSCAR was designed to evaluate six behavioural domains (communication, cooperation, coordination, monitoring/situation awareness, leadership and decision-making) for each of the three core team-members with leadership and coordination roles in a typical resuscitation team (such individuals commonly lead sub-teams). These were:

(1) The airway, ventilation and vascular access specialist, termed “Anaesthetist”, but could equally be a respiratory therapist, operating theatre practitioner, etc. – depending on local circumstances.
(2) The internal medicine specialist, termed “Physician”, but could equally be from critical care, surgery, etc.
(3) Senior nurse – either from the ward/floor area or arriving with the resuscitation team.

To minimise biases in the scoring and to ensure adequate inter-rater reliability in subsequent phases, “exemplar behaviours” were also defined. These are examples of optimum behaviours ideally seen when observing resuscitation teams’ interactions. For example, we would hope to arrive at a cardiac arrest and for the nurse looking after the patient to communicate a clear, concise account of exactly what has happened, and why the patient is in hospital, preferably using the “situation, background, assessment, recommendation” (SBAR) communication framework recommended by the Resuscitation Council (UK).^22^ An example of poor communication would occur when the nurse is unable to give any helpful information on arrival of the team; this would actively hinder resuscitation attempts. The exemplars were developed from the well-validated OTAS exemplars^16,23^ – but modified as required to ensure applicability to resuscitation (Table 1). The tool and exemplars were developed to measure behaviours seen within all members of the sub-teams. However, naturally, most of those looking at, for example, leadership qualities focused on the leader for each sub-team.

### 2.2 Phase 2 – face and content validation

The face and content validity of exemplars developed for each sub-team (anaesthetists, physicians, and nurses) were systematically assessed following standard recommendations^19^ by ten experts within the field of resuscitation (Online Appendix 1). To ensure content and face validation within and across specialties and minimise potential specialty-specific biases, each set of exemplars was rated by five experts within that speciality and five experts outside it. For example, the Anaesthetic behaviours were assessed by five anaesthetists, and five nurses or physicians. Each exemplar was rated for importance using a Likert scale of 1–4 (1 = of minor importance; 4 = of critical importance). Raters were also asked to make suggestions of additional exemplars, modifications of wording, or deletions, as they felt appropriate.

Content validity of exemplars was formally assessed further via computing a mean and standard deviation rating for each exemplar, one for the specialty experts (e.g., anaesthetists for anaesthetic exemplars) and one for the non-specialty experts (e.g., physicians and nurses for anaesthetic exemplars). Behaviours with a mean score of three or less (i.e., scored at or below the third quartile of the scale) were subsequently discussed by the development team (two anaesthetists and two psychologists with expertise in non-technical skills and tool development) and amended or discarded according to raters’ recommendations and opinions (Table 2).

### 2.3 Phase 3 – reliability assessment

Phase 3 aimed to assess the following features of OSCAR:

(a) Internal consistency
(b) Inter-rater reliability

Eight videos of cardiac arrest teams performing resuscitation simulations were watched by two expert clinical observers. They used OSCAR independently of each other to rate the Cardiac Arrest Teams performance. Four of the videos watched were simple cardiac arrests from a simulation training suite, and four were videos of unannounced *in situ* cardiac arrest simulations performed in a clinical hospital environment utilising the on-service cardiac arrest team for the day. These scenarios varied, from a massive post-partum haemorrhage on labour ward to a ruptured abdominal aneurysm in the radiology department. *In situ* simulations are part of our Hospital's continuous resuscitation training programme.

### 2.4 Statistical analysis

All data analyses were carried out using SPSS v. 18.0 (SPSS Inc., Chicago, IL, USA). Reliability in the form of internal consistency was assessed using Cronbach's α. Adequate internal consistency is typically demonstrated with Cronbach's α in the region of 0.70–0.90. The analysis identifies exemplars that should be removed to improve internal consistency; three exemplars were therefore removed.

After deletions were made from the tool following primary Cronbach's α analysis, the remaining exemplars were assessed for intraclass correlation (ICC) to demonstrate inter-rater reliability. Intraclass correlations of 0.70 or higher typically indicate adequate agreement in the scoring between independent raters.

## 3 Results

### 3.1 Phase 1 – review of evidence base and tool development

The result of this phase was an initial version of the OSCAR tool, which could then be face and content validated by resuscitation experts in Phase 2. This first iteration contained three behaviour exemplars for each team member (anaesthetist, physician, nurse) in each of the six behaviour domains. Therefore, a total of fifty-four different behaviour exemplars were assessed further.

### 3.2 Phase 2 – face and content validation

Thirty-nine of the fifty-four exemplars were deemed “critically important behaviours” by consensus of the resuscitation experts, with only fifteen of the fifty-four exemplars scoring mean values of three or less from the specialty expert or non-speciality expert group. The fifteen exemplars that were given low scores by either the specialty or non-specialty groups were reviewed by the tool development team (Table 2). Modifications were made in accordance with suggestions made by the experts, and opinions of the development team. As a result, the wording was modified in seven exemplars, four exemplars were deleted, and four were reviewed but not modified as they were felt by the development team to be important, and had been rated highly by one or other of the expert rating groups. In addition, wording was modified slightly for two exemplars that had been rated highly by both specialty and non-specialty teams, on the basis of suggestions made by these experts. Finally one new exemplar was added due to recommendations made by the experts. A total of eighteen changes were made.

### 3.3 Phase 3 – reliability assessment

Table 3 summarises the Cronbach's α coefficients in each behaviour domain for each of the three sub-teams (anaesthetists, physicians and nurses). Cronbach's α coefficient results range from 0.736 to 0.965, with fifteen of eighteen behaviours (83%) demonstrating very high internal consistency (Cronbach α > 0.80). Analyses dictated removal of three behaviour exemplars at this point (two removed from the anaesthetist group, one from the physician group). These were not necessarily behaviours that are unacceptable during resuscitation, but ones that were not consistently measurable. The three that were removed are listed below:

1. Co-operation: anaesthetist assists voluntarily with non-airway tasks if airway secure and more than one airway expert present.
2. Co-ordination: team members prepare drugs and equipment for anaesthetist (with or without instruction).
3. Decision making: timely and appropriate decisions by Physician regarding when to stop resuscitation attempts.

**Fig. 2:**
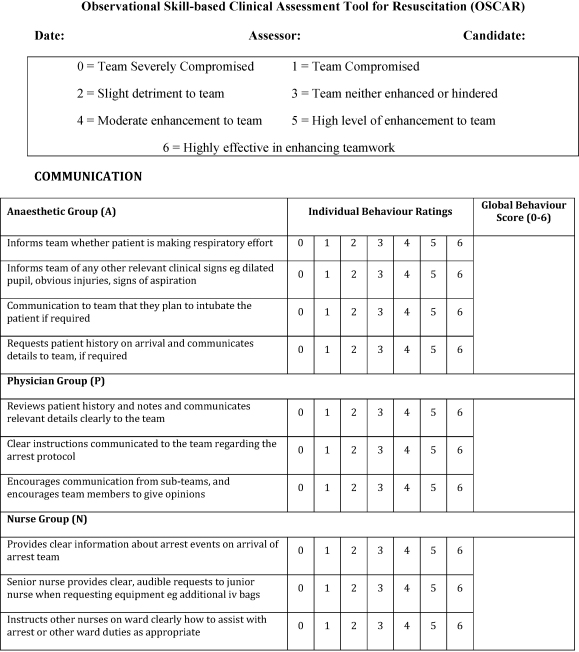

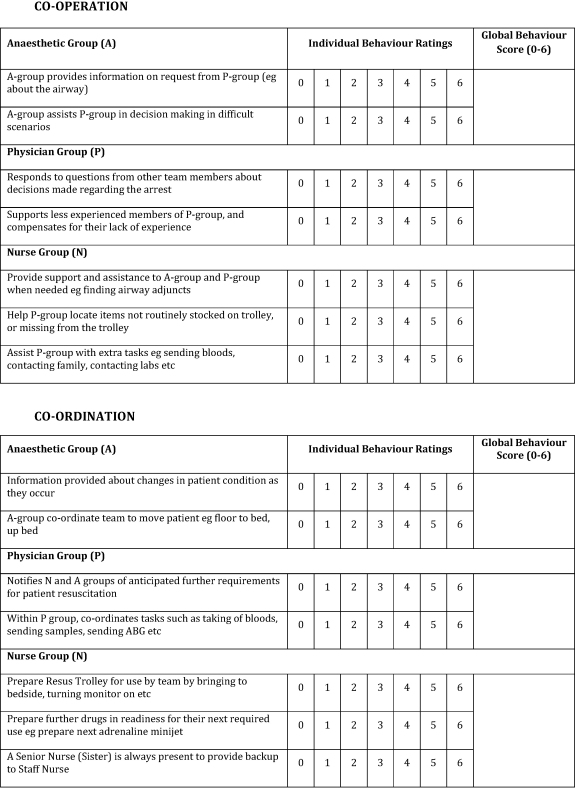

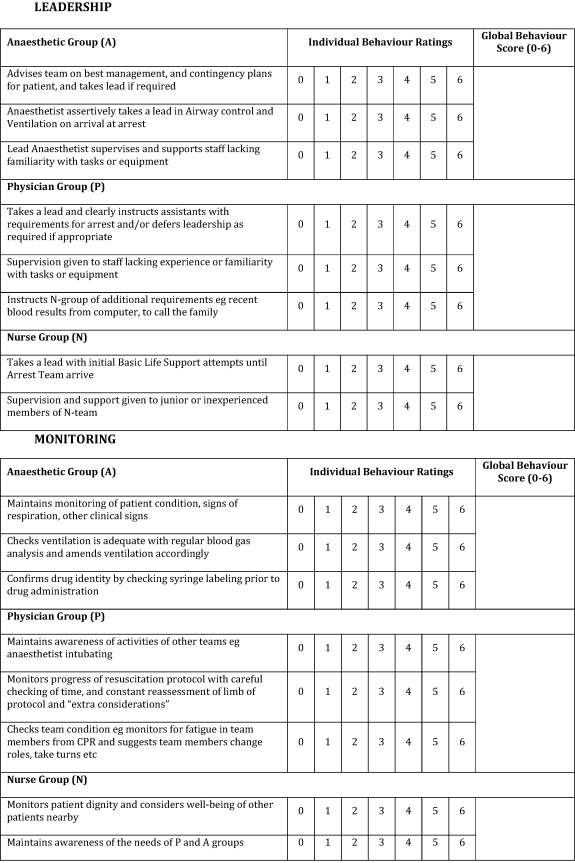

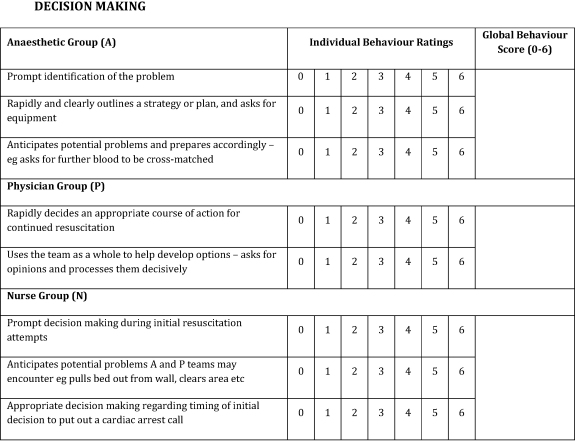
Final version of OSCAR tool Observational Skill-based Clinical Assessment tool for Resuscitation (OSCAR).

Intraclass correlations were subsequently calculated from the refined tool (Table 4). Intraclass correlations were strong and highly significant for all behaviours across all three subgroups, thereby indicating very good inter-rater agreement in the scoring of all the behaviours. The final version of OSCAR is shown in Fig. 2.

## 4 Discussion

The aim of the study reported here was to address the relative lack of tools for the assessment of non-technical skills in the context of resuscitation. Specifically, we sought to develop a tool that is feasible to use and psychometrically sound (reliable and valid). In doing so, our specific motivation was to enable us to measure and train non-technical skills, with systematic, evidence-based constructive feedback to emergency teams during mandatory simulation training.

We methodically developed the Observational Skill-based Clinical Assessment tool for Resuscitation (OSCAR). We developed OSCAR from existing well-validated instruments that have been developed for other contexts (OTAS, ANTS and NOTECHS)^3,16,17^ to ensure content validity and adequate coverage of evidence-based behaviours (Phase 1). We then undertook a thorough process of expert content validation leading to further tool amendments (Phase 2). Finally, we tested two forms of OSCAR reliability, internal consistency and inter-rater agreement, and empirically demonstrated more than adequate results in both. On this basis, we conclude that OSCAR is psychometrically robust, scientifically sound, and clinically relevant. This tool is intended for use by someone with experience in resuscitation, although prior experience in the use of behaviour assessment tools would not be required. It could be used in simulation centre training, or in a ward environment; simulated or real. The user would require some limited instruction in its use.

Recently, two other research groups have published tools similarly aimed at assessing non-technical skills in Resuscitation. The first of these is called the Team Emergency Assessment Measure (TEAM).^24^ This consists of eleven assessments of team performance rated on a Likert scale of 0–4, and a final overall performance score rated from 1 to 10, therefore a total of twelve points. Assessments are made in a variety of domains including communication, situation awareness, and team morale. A comparison of OSCAR with TEAM reveals overall similar behaviours being assessed and a similar development process. The tools do differ however: whereas TEAM assesses the entire team on twelve discrete points, OSCAR assesses each resuscitation team-member (Anaesthetist, Physician and Nurse) separately capturing six behaviours in detail within these subgroups–resulting in a total of forty-eight points assessed. We anticipate that whereas TEAM may be quicker for an assessor to use, OSCAR is likely to provide a more detailed and insightful breakdown of resuscitation team behaviours. In addition, OSCAR allows feedback to individual team members of their non-technical skills. Formal research comparison of the two instruments is now needed to delineate how much they overlap in practice.

The second is from a research group based in Denmark, who firstly identified the non-technical skills suitable for improving team performance in cardiac arrest teams, ^5^ and then developed checklists to be used on a course they developed to assess technical and behavioural aspects of cardiac arrest team performance.^25^ Their list of recommended behaviour categories, whilst given slightly different terms to ours, incorporates the same behaviour groups we have identified to assess. The assessment of behavioural markers assesses the behaviours of the team as a whole on a dichotomous scale (“yes” and “no”). In their discussion they acknowledge that other behaviour assessment tools are often scored using Likert-like scales, and that this gives the possibility of greater variability in assessment, but that they wanted a tool that was less complicated and easy to use. In a similar way to the “TEAM” tool discussed above, we feel that when compared with the tool developed by Andersen et al., OSCAR is likely to provide a more detailed breakdown of non-technical skills of individual team members, whilst we acknowledge it may be more complicated to use. A formal comparison of the tools is required.

Further research is also required to assess the utility and scope of OSCAR. First, we intend to use the tool to assess performance in real resuscitations. The study was limited to adult resuscitation and would need further development for a paediatric context, but we believe the basic underlying principle would be similar. We believe this would also apply in major trauma, which is a much more complicated clinical scenario, with further specialty groups involved, such as radiology, surgery, neurosurgery, and thus more vulnerable to a non-technical skills failure impairing performance.

We acknowledge that there has in the past been limited education of non-technical skills within clinical training curricula, although this is something that is gradually changing. The most recent version of the European Resuscitation Council Guidelines includes a section about education techniques, emphasising the importance of non-technical skills to improve resuscitation.^26^ We expect that resuscitation team members may or may not exhibit some of the skills captured by OSCAR. However, we anticipate that use of OSCAR during real and simulated resuscitation attempts (peri-arrest or full arrest) will enable identification of areas of weakness/opportunities for improvement in team members’ non-technical skills, as illustrated in Online Appendix 2. This in turn will enable us to facilitate post-arrest/scenario constructive feedback, and focussed training in these areas at a future date. We anticipate this will lead to an overall improvement in team performance at emergency events, which will ultimately translate into a subsequent reduction in the rate of errors and adverse events. We also hope that an increased awareness of non-technical skills in the emergency setting will have an indirect beneficial effect on those skills in the day-to-day setting. Targeted training to improve specific weaknesses in non-technical skills will in the long run lead to a flattening of hierarchy, which is well-known to improve the culture of patient safety.^27,28,29^

## 5 Conclusion

We have developed the Observational Skill-based Clinical Assessment tool for Resuscitation (OSCAR) for the assessment of non-technical skills in resuscitation teams. The tool has demonstrated face and content validity, feasibility, high internal consistency, and inter-rater reliability. We propose the use of this tool in simulation and real cardiac arrest resuscitation attempts to assess, guide and train non-technical skills to team members, thus striving to reduce rates of adverse events in these incident-prone circumstances and improve patient safety.

## Conflict of interest statement

S Brett is a co-author on the worksheet “Quality of life after resuscitation” in the 2010 guideline revision. He has a research grant from Carefusion, and consults for Pfizer and Baxter Healthcare.

No other conflict of interest is declared.

## Funding

This research was funded by a grant from the Wellcome Trust, UK. The funding source of the study had no role in the study design, data collection, data analysis, data interpretation, writing of the report, or the decision to submit for publication. S. Walker, C. Vincent and N. Sevdalis are affiliated with the Centre for Patient Safety and Service Quality at Imperial College Healthcare NHS Trust, which is funded by the UK's National Institute of Health Research. S. Brett wishes to acknowledge the support of the UK NIHR Comprehensive Biomedical Research Centre Scheme.

## Figures and Tables

**Table 1:** Illustration of how exemplar behaviours were modified from OTAS (operating room environment) for OSCAR (resuscitation environment).

Behaviour	Team member	Existing OTAS exemplar	New OSCAR exemplar
Communication	Anaesthetist	Provides update on patient condition and anything administered to patient	Informs team whether patient is making respiratory effort
	Physician	Requests and instructions to team communicated clearly and effectively	Clear instructions communicated to the team regarding the arrest protocol
	Nurse	Scrub Nurse provides clear and audible requests for provisions to charge nurse	Senior nurse provides clear, audible requests to junior nurse when requesting equipment, etc.
Co-operation	Anaesthetist	Anaesthetic group provided timely information on request from nurse group	Anaesthetic group provides information on request from physician group
	Physician	Responds to questions and request from nurse group	Responds to questions from other team members about decisions made regarding the arrest
	Nurse	Provide support and assistance to anaesthetic group when needed	Provide support and assistance to anaesthetic group and physician group when needed

**Table 2:** All exemplars for anaesthetists, physicians, and nurses with mean ratings by specialty experts (S) and non-specialty expert (N-S). Behaviours subsequently reviewed shaded in grey with initiating score.

Behaviour	Anaesthetists (A)			Physicians (P)			Nurses (N)		
	Exemplar	S	N-S	Exemplar	S	N-S	Exemplar	S	N-S
Communication	Informs team whether patient making respiratory effort	3.8	3.8	Reviews patient history and notes, and communicates details clearly to team	3.6	3.8	Provides clear information about arrest events on arrival of arrest team	3.8	3.8
	Informs team of any other relevant clinical signs	3.8	3.2	Clear instructions communicated to the team regarding arrest protocol	3.8	4	Senior nurse proved clear audible requests to junior nurse	3.6	3.8
	Communication to team that they plan to intubate the patient	4	3.8	Talks to the team to encourage communication from sub-teams	3.2	3	Instructs other nurses on ward clearly how to assist arrest, or other ward duties	3.2	2.8
Co-operation	A-group provides information on request from P-group	3.8	3.8	Responds to questions from other team members about decisions made	3.4	3.8	Provide support and assistance to A-group and P-group when needed	3.6	3.8
	A-group assists P-group in decision mating in difficult scenarios	4	3.8	Provides assistance to N-group in setting up fluid giving sets, etc.	2.2	2.8	Help P-group locate items required not routinely stocked or missing from trolley	3.8	3.8
	Assists voluntarily with non-airway tasks if airway secure and >1 A-group present	3.4	3.4	Supports less experienced members of P-group_,_ and compensates for them	3	3.6	Assist P-group with extra tasks, e.g. blood bottle labeling	2.8	3.2
Co-ordination	Junior anaesthetist prepares drugs and equipment for senior	3.4	2.8	Notifies N and A groups of anticipated further requirements for resuscitation	3.4	3.6	Prepare resus trolley for use by team by bringing to bedside	3.4	3.8
	Information provided about changes in patient condition as they occur	4	3.6	Assists in transfer of patient	2	3.2	Prepare further drugs, in readiness for their next required use, e.g. adrenaline	3.8	2.8
	A-group co-ordinate team to move patient	3.8	3.2	Within P-group co-ordinates tasks such as taking of bloods, etc.	3.4	3.6	A senior nurse is always present to provide back-up to staff nurse	3.6	3.4
Leadership	Advises team on best management and contingency plans for patient	3.6	3.2	Takes a lead and clearly instructs assistants with requirements for arrest	4	3.8	Takes a lead with initial basic life support attempts until arrest team arrives	3.8	4
	Anaesthetist assertively takes a lead in Airway Control on arrival	3.8	3.6	Supervision given to staff lacking experience or familiarity with tasks	3.4	3.4	Assertive in controlling noise and distractions during resuscitation	3.2	2.8
	Lead Anaesthetist supervises and supports staff lacking familiarity	3.6	3.2	Instructs N-group of additional requirements, e.g. blood results	2.6	3	Supervision and support given to junior or inexperienced members of N-team	3.2	3.2
Monitoring	Maintains monitoring of patient condition	4	3.6	Monitors progress of other teams	2.6	3.6	Monitors progress closely, and documents drugs given carefully	3.2	3
	Checks ventilation adequate with ABG analysis, amends ventilation accordingly	3.6	3.2	Monitors progress of resuscitation protocol, checking times, etc.	3.6	4	Monitors patient dignity and considers well-being of other patients nearby	3	2.8
	Checks all drugs, monitoring, and equipment prior to use	3.4	3	Checks team condition, e.g. monitors for fatigue	3.2	3.6	Monitors the needs of P and A groups	3.2	3
Decision-Making	Prompt identification of the problem	4	3.4	Rapidly decides an appropriate course of action for resuscitation	3.8	4	Prompt decision making during initial resuscitation attempts	4	4
	Rapidly and clearly outlines a strategy or plan, and asks for equipment	4	3.6	Uses the team as a whole to help develop options	3.6	3.4	Anticipates potential problems A and P teams may encounter	3.6	3.2
	Anticipates potential problems and prepares accordingly	3.6	3.4	Timely and appropriate decision regarding when to stop if unsuccessful	3.8	4	Appropriate decision making regarding timing of when to put out arrest call	3.8	4

**Table 3:** Internal consistency reliability (Cronbach alpha coefficients) across all OSCAR behaviours and rated subgroups.

Team subgroup	Behaviour					
	Communication	Co-operation	Co-ordination	Leadership	Monitoring	Decision making
Anaesthetists	0.951	0.745	0.771	0.952	0.814	0.965
Physicians	0.925	0.874	0.855	0.889	0.949	0.933
Nurses	0.874	0.948	0.852	0.797	0.736	0.875

*Note*: Cronbach alpha coefficients can range between 0 and 1, with higher coefficient indicating better internal consistency of the scoring. Coefficients of ≥0.70 are typically considered as very good.

**Table 4:** Inter-rater reliability (Intraclass Correlations) across all OSCAR behaviours and rated subgroups.

Team subgroup	Behaviour mode						
	Communication	Co-operation	Co-ordination	Leadership	Monitoring	Decision making	Overall
Anaesthetists	0.835 (*N* = 32)	0.805 (*N* = 16)	0.876 (*N* = 16)	0.718 (*N* = 24)	0.664 (*N* = 24)	0.787 (*N* = 24)	0.767 (*N* = 136)
Physicians	0.761 (*N* = 24)	0.744 (*N* = 16)	0.743 (*N* = 16)	0.836 (*N* = 24)	0.833 (*N* = 24)	0.895 (*N* = 16)	0.809 (*N* = 120)
Nurses	0.814 (*N* = 24)	0.652 (*N* = 24)	0.890 (*N* = 24)	0.744 (*N* = 16)	0.823 (*N* = 16)	0.911 (*N* = 24)	0.807 (*N* = 128)

*Note*: Intraclass correlation coefficients can range between 0 and 1, with higher coefficient indicating better agreement between two or more assessors. Coefficients of ≥0.70 are typically considered as very good. In the table above, all coefficients are significant at *p* < 0.001.
