# Supplementary material for: Observational Skill-based Clinical Assessment tool for Resuscitation (OSCAR): Development and validation
Source: Resuscitation. 2011 Jul;82(7):835–44. doi: 10.1016/j.resuscitation.2011.03.009 (PMC3121958; doi:10.1016/j.resuscitation.2011.03.009)
Supplement: Supplementary file 1 [file mmc1.doc]

Resumen

*Objetivo*: El objetivo de este estudio fue evaluar la necesidad de evaluar y entrenar trabajo de equipo y destrezas no técnicas en el contexto de la resucitación. Específicamente, buscamos desarrollar una herramienta que sea factible de usar evaluar psicométricamente para evaluar comportamientos de equipo durante intentos de resucitación de paro cardiaco.

*Métodos*: Para asegurar validez, confiabilidad, y factibilidad, se desarrolló la herramienta Herramienta de Evaluación clínica basada en destrezas observacionales (OSCAR)

*Resultados*:

*Conclusión*:≤≥±≠μ®™ ± *p* = ^o^C

© 2011 Publicado por Elsevier Ireland Ltd.

*Palabras clave*: Paro cardiaco extrahospitalario; Reanimación cardiopulmonar (RCP); Adultos jóvenes; epidemiología; Servicio de ambulancias; Australia; registro de paro cardiaco
